# Basis for using thioredoxin as an electron donor by *Schizosaccharomyces pombe* Gpx1 and Tpx1

**DOI:** 10.1186/s13568-022-01381-2

**Published:** 2022-04-11

**Authors:** Fawad Ahmad, Muhammad Faizan Latif, Ying Luo, Ying Huang

**Affiliations:** Jiangsu Key Laboratory for Microbes and Genomics, Department of Microbiology, School of Life Sciences, Nanjing Normal University, 1 Wenyuan Road, Nanjing, 210023 China

**Keywords:** *Schizosaccharomyces pombe*, Reactive oxygen species (ROS), GSH peroxidase, Trx peroxidase

## Abstract

Glutathione (GSH) peroxidases (GPxs or GSHPx) and thioredoxin (Trx) peroxidases (TPxs) are two classes of peroxidases that catalyze the reduction of peroxides. GPxs and TPxs generally use GSH or Trx, respectively, to recycle the oxidized cysteine (Cys) residue in the protein. However, it is unclear why unlike human GPxs, the *Schizosaccharomyces pombe* Gpx1 (spGpx1) prefers Trx over GSH for recycling of the active-site peroxidatic Cys residue. Here, we compared spGpx1 and *S. pombe* Tpx1 (spTpx1) protein sequences with those of their respective homologs in *Saccharomyces cerevisiae* and humans. Our analysis revealed that like spTpx1, spGpx1 contains a pair of conserved Cys residues (Cys36 and Cys82). These two conserved Cys residues are named peroxidatic and resolving Cys residues, respectively, and are found only in GPxs and TPxs that prefer Trx as an electron donor. Our analysis suggested that Cys36 and Cys82 in spGpx1 are most likely to form a disulfide bond upon oxidation of Cys36. Molecular modelling predicted that a conformational change might be required for the formation of this disulfide bond. Evolutionary analysis suggested that fungal GPxs and TPxs are related by divergent evolution from a common ancestor. Our analyses support a prediction that while spGpx1 and spTpx1 are phylogenetically and functionally different, they evolved from a common ancestor and use a similar mechanism for recycling of the active-site peroxidatic Cys residue.

**Supplementary Information:**

The online version contains supplementary material available at 10.1186/s13568-022-01381-2.

## Key points

- spGpx1 contains two conserved Cys residues (Cys36 and Cys82), which may form a disulfide bond upon oxidation and the reduction of this disulfide bond is most likely to be mediated by Trx *in vivo*.
- Fungal GPxs and TPxs evolved from a common ancestor.

**Supplementary Information:**

The online version contains supplementary material available at 10.1186/s13568-022-01381-2.

## Introduction

During normal cellular metabolic processes, all organisms produce reactive oxygen species (ROS), such as superoxide (⋅O_2_^−^), hydroxyl radical (⋅OH), and hydrogen peroxide (H_2_O_2_**)** (Winterbourn 2008, Kong and Chandel 2018). In addition, ROS can also be generated when organisms are exposed to environmental agents such as ionizing radiation, UV light, chemicals, and metals. Under physiological conditions, a certain level of ROS can act as signaling molecules in the maintenance of physiological functions (Schieber and Chandel 2014, Sies and Jones 2020). However, the toxic levels of ROS cause damage to lipids, proteins and DNA, and are implicated in aging and numerous diseases, including neurodegenerative diseases and cancer.

To deal with ROS toxicity, cells have evolved mechanisms such as production of enzymatic and nonenzymatic antioxidant defenses (Do et al. 2019). Antioxidant enzymes include superoxide dismutase, which catalyzes the dismutation of superoxide anions into oxygen and H_2_O_2_, catalase that degrades the conversion of H_2_O_2_ into water and oxygen, and thiol-dependent peroxidase that uses redox-active cysteines (Cys) or selenocysteine (Sec) to reduce H_2_O_2_ or organic hydroperoxides (ROOR) to H_2_O or alcohols (Nordberg and Arnér 2001, Tairum et al. 2012). Nonenzymatic antioxidant defenses include glutathione (GSH), ascorbic acid, glutathione-S-transferase (GST) and metallothionein.

Glutathione (GSH) peroxidases (GPxs or GSHPx) are thiol peroxidases that use GSH or thioredoxin (Trx) as the electron donor (Brigelius-Flohé and Maiorino 2013). GPxs can be classified into the selenium-dependent GPx (Se-GPx) that reduces organic as well as inorganic peroxides, and non-selenium-dependent GPx (non-Se-GPx) that reduces only organic peroxides (Xia et al. 2016). Mammals have both Se-GPxs and non-Se-GPxs, whereas bacteria, fungi and plants possess only Se-GPxs.

*Saccharomyces cerevisiae* has 3 GPxs [scGpx1, scGpx2 and scGpx3 (also known as Hyr1 or scOrp1)]. scGpx2 and scGpx3 prefer Trx over GHS as an electron donor. Although scGpx1 uses GSH and Trx almost equally as the electron donor in vitro, Trx plays a crucial role in the reduction of spGpx1 in vivo (Ohdate et al. 2010, Ukai et al. 2011). The three proteins are localized in the peroxisome, mitochondrial, and cytosol, respectively (Ohdate and Inoue 2012). Unlike scGpx1 and scGpx2, scGpx3 also functions as the major H_2_O_2_ sensor in the cytosol. After oxidized by H_2_O_2_, the Cys36 of scGpx3 forms an intermolecular disulfide bond with the Cys598 of scYap1, the central regulator of oxidative stress response in *S. cerevisiae*. This disulfide bond is rearranged to form an intramolecular disulfide bond in scYap1, leading to the activation of scYap1 (Delaunay et al. 2002). In addition, an isoform of scGpx3 is localized in mitochondria and facilitates oxidative protein folding (Kritsiligkou et al. 2017). In contrast to most other organisms that contain several GPxs, *Schizosaccharomyces pombe* has only a single GPx (spGpx1). Like *S. cerevisiae* GPxs, spGpx1 prefers Trx over GSH for electron donor (Lee et al. 2008, Paulo et al. 2014). Consistent with the view that the human genome shows a tendency to expand the number of ROS-related proteins compared to fungi, humans have 8 GPxs including 5 selenoproteins (hsGPx1-hsGPx4, and hsGPx6) and 3 non-selenoproteins (Matoušková et al. 2018).

The reduction of peroxide by Se-GPxs involves the formation of several intermediate modifications to the Sec active site (Lubos et al. 2011). After reacting with peroxide, the selenol group (Se-H) at the active site is oxidized to selenenic acid (Se-OH), and one molecule of GSH then reduces Se-OH and forms glutathiolated selenol (Se-GS). Subsequently, another molecule of GSH reduces Se-GS, and simultaneously generates the oxidized form of GSH disulfide (GSSG), which is reduced to regenerate GSH by GSH reductase using NADPH as an electron donor. The active sites of non-Se-GPxs contain a Cys residue instead of a Sec residue. In contrast, the reduction of peroxide by non-Se-GPxs involves the formation of an intramolecular disulfide bond between two Cys residues. The first redox-active Cys residue, designated the peroxidatic Cys is oxidized by peroxide, yielding sulfenic acid (Cys-SOH). This oxidized Cys residue then forms a disulfide bond with another Cys residue, designated the resolving Cys. This disulfide bond is reduced by the Trx system, which is composed of NADPH, Trx reductase (TrxR), and Trx.

Peroxiredoxins (Prxs or Prdxs) are a family of thiol peroxidases that catalyze the reduction of H_2_O_2_, organic hydroperoxides, and peroxynitrite (Wood et al. 2003a, b, Wood et al. 2003a, b, Perkins et al. 2015, Rhee 2016, Bolduc et al. 2021). Based on the number of active cysteine residues, Prxs can be categorized into 1-Cys and 2-Cys Prxs. All 2-Cys Prxs share the similar catalytic mechanism. First, a redox-active Cys residue (called the peroxidatic Cys) is oxidized to a sulfenic acid (Cys-SOH) by either H_2_O_2_ or organic hydroperoxides. Second, the sulfenic acid forms a disulfide bond with another redox-active Cys (called the resolving Cys) of the other subunit of the Prx homodimer (typical 2-Cys Prxs), or with the C-terminal resolving Cys of the same monomer (atypical 2-Cys Prxs). Last, this resulting disulfide bond is normally reduced by thioredoxin. Thus 2-Cys Prxs are also named Trx peroxidases (TPxs). For 1-Cys Prxs, the sulfenic acid is directly reduced to thiol due to no nearby Cys to form a disulfide bond.

Prxs are found in both eukaryotic and prokaryotic organisms. *S. cerevisiae* has five Prxs (scTsa1, scTsa2, scAhp1, scPrx1, and scDot5) (Kim et al. 2010). Mammalian cells have 6 Prxs: 4 typical 2-Cys Prx (huPrx1-huPrx4), 1 atypical 2-Cys Prx (huPrx5) and 1 1-Cys Prx (huPrx6) (Rhee and Kil 2017). *S. pombe* contains 3 Prxs (spTpx1, spPmp20, and spBcp1). spTpx1 is a 2-Cys Prx that functions as the main H_2_O_2_ scavenger during aerobic growth in *S. pombe* (Jara et al. 2007, Paulo et al. 2014). spTpx1 also functions as the H_2_O_2_ sensor that transduces the redox signal to the transcription factor spPap1, which a homolog of scYap1 (Paulo et al. 2014). Upon exposure to lower levels of H_2_O_2_, Cyc48 in spTpx1 is oxidized to sulfenic acid, which can form a disulfide bond with Cyc. The reduction of this labile disulfide bond is mediated by the Trx system (Jara et al. 2007, Lu and Holmgren 2014). The sulfenylated spTpx1 can activate spPap1 by inducing the formation of an intramolecular disulfide bond in spPap1, which then translocates from cytoplasm to nucleus, where it activates genes required for adaptation to oxidative stress (Quinn et al. 2002, Calvo et al. 2013). However, at high levels of oxidants, the sulfenylated spTpx1 can be further oxidized to the sulfinic acid (SO_2_H) or sulfonic acid (SO_3_H) forms, resulting in loss of its peroxidase activity and prevention of Pap1 activation (Bozonet et al. 2005, Vivancos et al. 2005). The hyperoxidized spTpx1 can be reduced to restore the peroxidase activity by sulfiredoxin spSrx1. The functions of spPmp20 and spBcp1 are unclear.

In this study, we conducted a bioinformatics analysis to understand the basis for why both spGpx1 and spTpx1 use Trx as an electron donor. Our analyses revealed that spGpx1 harbors two conserved Cys residues that are found in other GPxs and TPxs that use Trx as the electron donor. Our analysis suggests that like other Trx-dependent GPxs and TPxs, spGpx1 may also form a disulfide bond between Cys36 and Cys82 upon oxidation. Our phylogenetic study suggests that fungal GPxs and TPxs descended from a common ancestor.

## Methods

### Proteins data set and sequence analyses

Full-length sequences of the spGpx1 and spTpx1 were obtained from PomBase. We used the spGpx1 and spTpx1 protein sequences as queries in BLAST **(**Basic Local Alignment Search Tool) searches against human genome databases and fungal genome databases, including the National Center for Biotechnology Information (NCBI), using default parameters, to identify candidate GPxs and TPxs. All protein sequences were retrieved by using the cut-off *E*-value of 0.05. The UniProt Knowledgebase (UniProtKB) was used to check the presence of the conserved active site in the candidate proteins. The candidate proteins were then examined for the existence of the conserved domains using the Simple Modular Architecture Research Tool (SMART) (Letunic and Bork 2018). Multiple EM for Motif Elicitation (MEME Suite) Version 5.4.1 was used to identified the conserved sequence motifs using default parameters (Peng et al. 2018). The web-based prediction program WoLF PSORT was used to predict protein subcellular localization (Horton et al. 2007).

### Homology prediction of the three-dimensional structure of spGpx1 by I-TASSER

The structure of spGpx1 was predicted by the I-TASSER server (IterativeThreadingAssemblyRefinement-https://zhanglab.ccmb.med.umich.edu/I-TASSER/). The sequence was uploaded in FASTA format and output result was downloaded in PBD format. C-score was measured for the spGpx1 model and the lower rank model, which has a high C-score was selected as a better quality structure and C-score is confidence score for the model (Zhang 2008). UCSF Chimera was used for image visualization and structural analysis.

### Phylogenetic analysis

To construct a phylogenetic tree, a multiple amino acid sequence alignment was generated with the MUSCLE algorithm included in the Molecular Evolutionary Genetics Analysis (MEGA X) software (version 10.1.8) (Kumar et al. 2018). The phylogenetic tree was constructed using the maximum-likelihood method and the Jones-Taylor-Thornton (JTT) model in MEGA X, with 1000 bootstrap replications.

### Bayesian evolution analysis

To estimate the evolutionary rates of candidate fungal GPxs and TPxs, the Bayesian evolutionary tree was constructed using Beast v1.10.4 (Zhang and Drummond 2020). BEAST XML files were generated for GPxs and TPxs by using BEAUti software which is available in the BEAST package. The substitution model was based on BLOSUM62, and the 4-category gamma model was used for the site-heterogeneity model. A strict clock and constant-size coalescent tree prior were employed. The Markov Chain Monte Carlo (MCMC) sampling algorithm was used to estimate statistical confidence. BEAST analysis was run, and the tree was viewed by using the software FigTree v.1.4,4 (http://tree.bio.ed.ac.uk/software/figtree/). Tracer v1.7.2 was used to calculate substitution per amino acid per year was calculated as well (Rambaut et al. 2018).

## Results

### spGpx1 harbours two conserved Cys residues that are likely to form an intramolecular disulfide bond

We conducted BLAST homology analyses using the spGpx1 protein sequence against the human genome database and fungal genome databases as part of our attempts to investigate the structural and functional diversity of GPxs. For further analysis, we chose 12 GPxs: 1 from *S. pombe* (spGpx1), 3 from *S. cerevisiae* (scGpx1, scGpx2 and scGpx3) and 8 from humans (hsGPx1-hsGPx8). spGpx1 is more similar to *S. cerevisiae* GPxs than to human GPxs (Table 1). It shares the highest similarity with scGpx3 (71% identity). To examine the evolutionary relationships among these GPxs, the phylogenetic tree of GPxs was constructed based on the similarity, using the maximum-likelihood method (Additional file 1: Fig. S1). Our analysis suggested that spGpx1 is most closely related to scGpx3.

**Table 1:** Sequence comparison of spGpx1 to *S. cerevisiae* and human GPxs

	spGpx1	
	Identity (%)	Similarity (%)
scGpx1	53	62
scGpx2	64	75
scGpx3	71	77
hsGPx1	28	36
hsGPx2	31	40
hsGPx3	27	33
hsGPx4	32	40
hsGPx5	29	36
hsGPx6	25	33
hsGPx7	29	37
hsGPx8	29	36

**Fig. 1:**
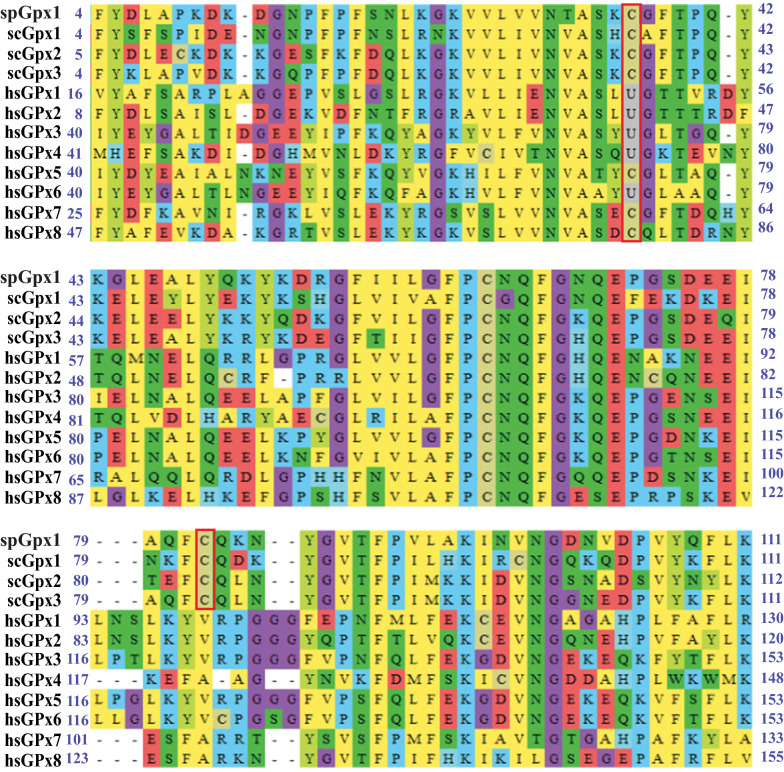
Multiple sequence alignment of sequences of the GSHPx domain in *S. pombe*, *S. cerevisiae* and human GPxs using the MEGA_X_10.1.8 software. The GSHPx domain was identified by searching the SMART database. The two characteristic residues (one Cys/Sec residue and one Cys residue) are boxed. Amino acid residues are highlighted according to the biochemical properties of the amino acid residues

To understand the structural and functional diversity of GPxs, we performed multiple sequence alignment of *S. pombe*, *S. cervisiae* and human GPxs. Multiple sequence alignment revealed the existence of a conserved Cys/Sec residue in all selected GPxs (Cys in spGpx1, scGpx1, scGpx2, scGpx3, hsGPx5, hsGPx7 and hsGPx8, and Sec in hsGPx1-hsGPx4 and hsGPx6). This Cys/Sec residue in GPxs has been demonstrated to be oxidized by peroxide (Delaunay et al. 2002, Tanaka et al. 2005, Ohdate et al. 2010). Our analysis also revealed an exclusively conserved Cys residue (Cys82 in spGpx1) in *S. pombe* and *S. cerevisiae* GPxs (Fig. 1). It has been shown an intramolecular disulfide bond formed by these two Cys residues is required for the peroxidase activity of the *S. cerevisiae* GPxs (Delaunay et al. 2002, Tanaka et al. 2005, Ohdate et al. 2010).

**Fig. 2:**
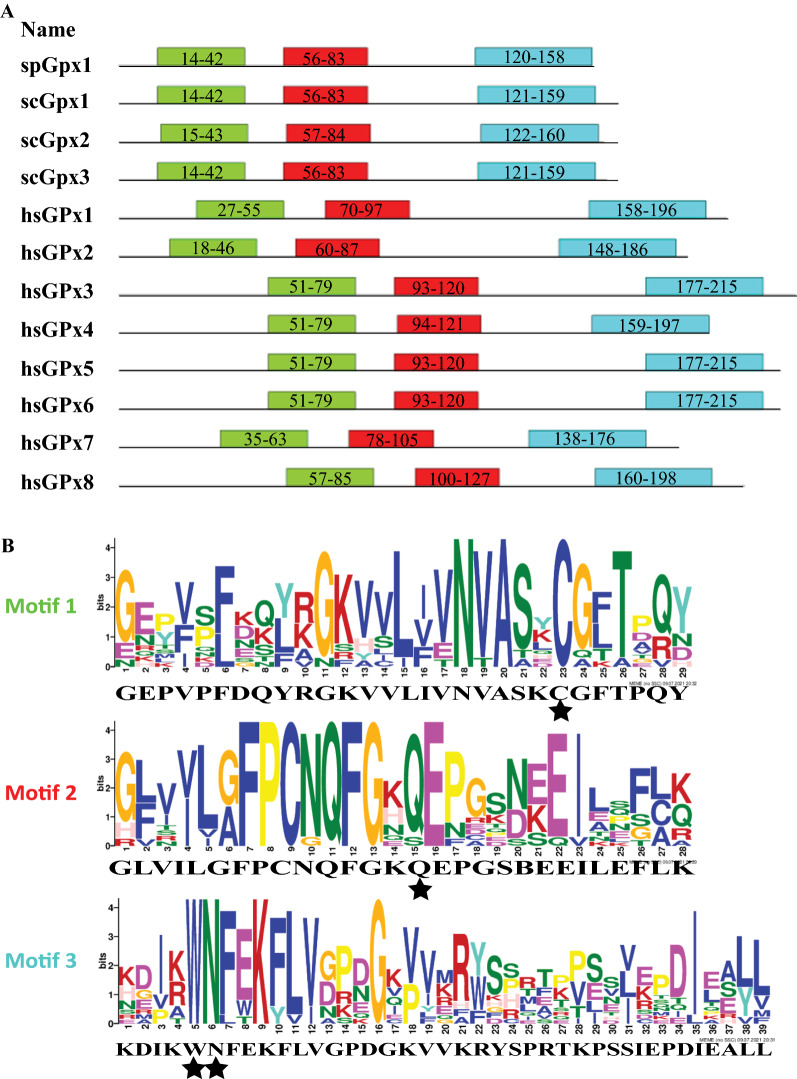
spGpx1 and its homologs contain 3 conserved motifs. **A** Locations of the motifs in GPxs. The motifs were detected by the MEME Suite (Version 5.4.1). Motifs 1-3 are labelled in green, red and blue, respectively. **B** Sequence logos for Motifs 1-3. The sequence logos of the motifs derived from *S. pombe*, *S. cerevisiae* and human GPxs were generated using the MEME Suite. The height of each amino acid indicates the level of conservation at that position. Amino acid residues are highlighted according to the biochemical properties of the amino acid residues. The catalytic residues Cys, Gln, Trp and Asn (Tosatto et al. 2008) are indicated by stars

Using the SMART database, we found that *S. pombe*, *S. cerevisiae* and human GPxs all contain the GSHPx domain that is involved in catalyzing the reduction of peroxide (Fig. 1). Using the MEME Suite, we identified 3 highly conserved motifs in these GPxs (Fig. 2). These three motifs are important for catalysis as they contain residues that participate in catalysis (Fig. 2).

### Homology modelling of spGpx1

**Fig. 3:**
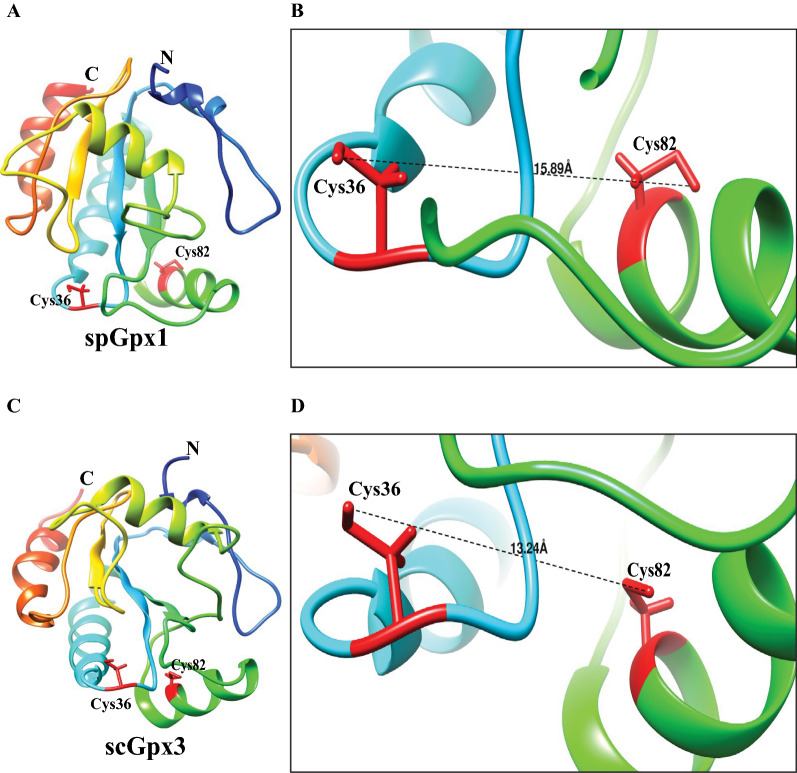
Homology model for spGpx1. **A** The ribbon diagram of the predicted structure of spGpx1 generated by using I-TASSER. **B** Close-up view of candidate peroxidatic and resolving Cys residues in spGpx1. The distances between the two Cys residues are marked as the dotted line. **C** Th ribbon diagram shown the structure of scGxp3. **D** Close-up view of the peroxidatic and resolving Cys residues in scGxp3

We carried out homology modelling of spGpx1 to determine whether the two conserved cysteines (Cys36 and Cys82) are in close proximity. Because spGpx1 shares the highest sequence similarity with scGpx3, whose structure has been determined (Zhang et al. 2008), we selected the structure of scGpx3 to build a three-dimensional model of spGpx1 using the online tool I-TASSER. The predicted spGpx1 structure is shown in Fig. 3. In the structure of spGpx1, Cys36 and Cys82 are in a distant of 15.89 Ǻ and is thus not in the close proximity to form a disulfide bond. Similarly, Cys36 and Cys82 are in a distant of 13.24 Ǻ in the scGpx3 structure. This analysis suggests that a conformational change is required to form a disulfide bond between these two Cys residues upon oxidation.

### spTpx1 contains N-terminal AhpC/TSA domain and C-terminal 1-cysPrx C domain.

**Fig. 4:**
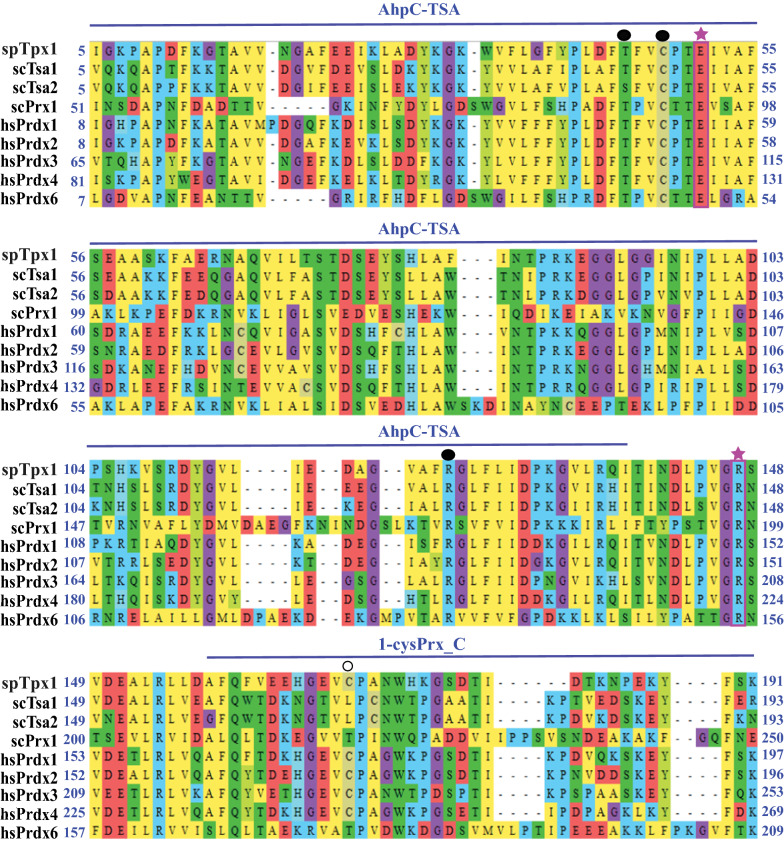
Multiple sequence alignment of TPxs from *S. pombe*, *S. cerevisiae* and humans. The alignment was constructed using the MEGA_X_10.1.8 software. The N-terminal AhpC-TSA domain and C-terminal 1-Cys Prx_C domain which are characteristic of Prxs were identified by searching the SMART database. The catalytic triad (Thr/Ser-Cys-Arg), the resolving residue and the Glu and Arg residues involved in Prx-Trx interaction are indicated by filled cycles, open circles and stars, respectively. Amino acid residues are highlighted according to the biochemical properties of the amino acid residues

A BLAST search using the spTpx1 protein sequence as a query revealed that spTpx1 homologs are present in *S. cerevisiae* and humans. Three spTpx1 homologs (scTsa1, scTsa2, and scPrx1) were found in *S. cerevisiae*, and five (hsPrdx1-hsPrdx4 and hsPrdx6) were found in humans. spTpx1 shares the highest sequence similarity (66% identity) with hsPrdx2 (Table 2). Phylogenetic analysis by maximum likelihood revealed that hsPrxd2 and scTsa1 were the most similar proteins to spTpx1 (Additional file 1: Fig. S2). SMART database searches showed that spTpx1 and representative Tpx1 homologs have an N-terminal AhpC/TSA domain and the C-terminal 1-cys Prx C domain, which are characteristic of Prxs (Fig. 4).

**Table 2:** Sequence comparison of spTpx1 to *S. cerevisiae* and human TPxs

	spTpx1	
	Identity (%)	Similarity (%)
scTsa1	64	71
scTsa2	60	69
scPrx1	24	31
hsPrdx1	60	70
hsPrdx2	66	76
hsPrdx3	45	55
hsPrdx4	42	51
hsPrdx6	26	36

Multiple sequence alignment of spTpx1 and its homologs in *S. cerevisiae* and humans (Fig. 4) revealed that except for scPrx1 and hsPrdx6, which has one-conserved Cys residue, all Prxs examined contain two characteristic Cys residues; an absolutely conserved peroxidatic Cys (Cys48 in scTsa1) and one highly conserved resolving Cys (Cys146 in scTsa1). Notably, the second Cys residues from spTpx1 and hsPrdx1-hsPrdx4 are not aligned with those in scTsa1 and scTsa2. The active sites of Prxs contain a catalytic triad composed of a peroxidatic Cys, a Thr (or a Ser) and an Arg (Tairum et al. 2016). This catalytic triad (Thr45-Cys48-Arg124 in ScTsa1) are found in spTpx1 and all other Prxs examined (Fig. 4). Glu51 and Arg147 in scTsa1 that are important for the scTsa1-Trx interaction are absolutely conserved in all Prxs examined (Tairum, de Oliveira et al. 2012).

Like scTsa1 and scTsa2, spTpx1 is a typical 2-Cys Prx that uses Trx to reduce the disulfide bond in Prxs. The reductant responsible for the reduction of the disulfide bond in 1-Cys Prx in vivo has not been definitively resolved. Candidate reductants include ascorbate, Trx, Trx reductase, glutaredoxin and GSH (Monteiro et al. 2007, Shadel Gerald and Horvath Tamas, 2015, Pedrajas et al. 2016).

### spGpx1 and spTpx1 are probably related via divergent evolution from a common ancestor

**Fig. 5:**
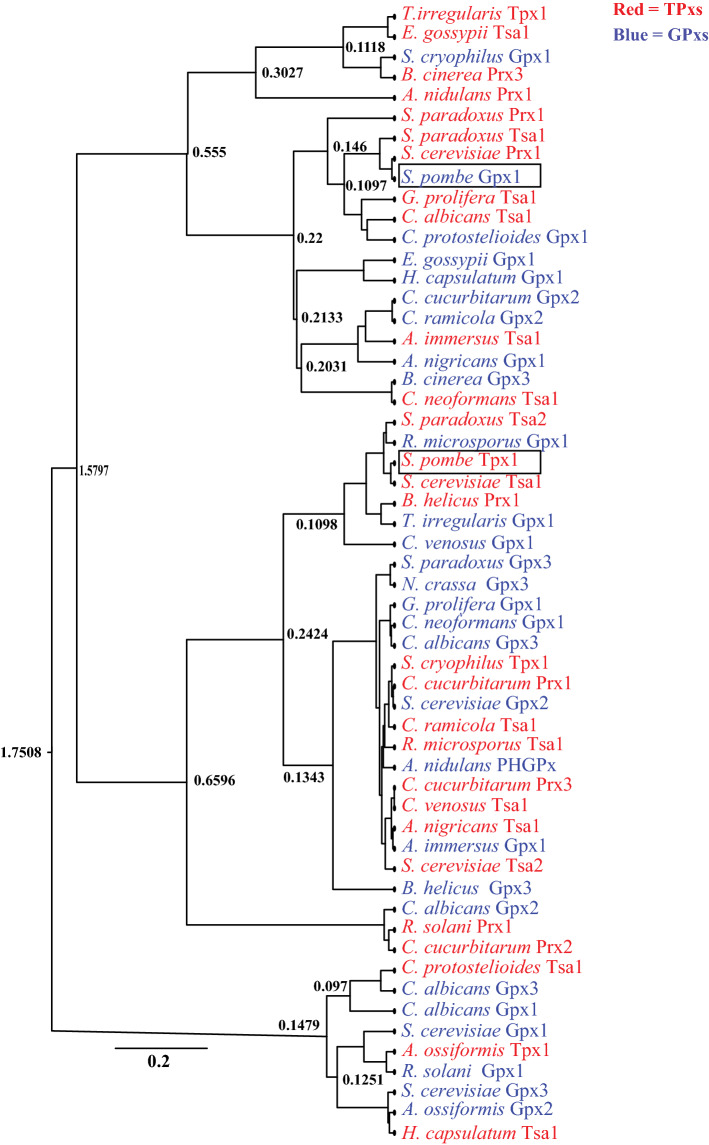
Bayesian evolutionary tree of fungal GPxs and TPxs using BEAST analysis. GPxs are colored in blue, and TPxs are colored in red. The strict clock model was used in the analysis. The reliability of nodes in the phylogeny was assessed using the MCMC algorithm. Bayesian posterior probabilities are shown at the nodes. The scale bar suggests that each site has 0.2 amino acid substitutions. The accession numbers for the proteins are provided in Tables S1 and S2

A Bayesian evolutionary analysis was performed using a data set composed of 28 GPxs and 28 TPxs belonging to taxonomically diverse fungi to determine the evolutionary relationships among fungal GPxs and TPxs (Additional file 1: Table S1 and S2). The evolutionary tree suggested that both fungal GPxs and TPxs share a common ancestor (Fig. 5). To estimate the mutation rate, first, we used the total 56 GPxs and TPxs. The mean substitution rate estimated for GPxs and TPxs using strict and relaxed clock models was 28.73076 and 32.2599 substitution/aa/year, respectively. Next, 28 GPXs were used to estimate the mutation rate, the evolutionary rate for the GPxs with the strict clock model calculated was 35.9718 substitutions/aa/year, whereas with the relaxed clock model, it was 36.9963 substitutions/aa/year. Furthermore, the mean evolutionary rate for the 28 TPxs was 12.6302 substitutions/aa/year with the strict clock model, and was 12.5194 substitutions/aa/year with the relaxed clock model. Our results showed that the TPxs have a relatively low substitution rate compared to GPxs (35.9718 substitutions/aa/year). However, the mean substitution rate for GPxs was relatively higher (35.9718 substitutions/aa/year) compared with GPXs/TPXs (28.73076 substitutions/aa/year) (Table 3).

**Table 3:** Evolutionary rate analysis of fungal GPXs and TPXs

Proteins	Demographic/clock model	Mean rate (substitutions/aa/yr)	95% high posterior density	Number of proteins
GPxs/TPxs	Gamma/strict clock	28.73076	[18.7884, 37.9342]	56
	Gamma/uncorrelated relaxed clock	32.2599	[18.9417, 47.9533]	56
	Gamma/fixed local clock	28.1658	[19.1689, 37.7794]	56
GPxs	Gamma/strict clock	35.9718	[18.2799, 58.5151]	28
	Gamma/uncorrelated relaxed clock	36.9963	[14.6074, 66.1576]	28
	Gamma/fixed local clock	35.6018	[18.5911, 58.2634]	28
TPxs	Gamma/strict clock	12.6302	[7.0483, 19.4014]	28
	Gamma/uncorrelated relaxed clock	12.5194	[7.0702, 19.6361]	28
	amma/fixed local clock	12.7281	[7.2035, 19.535]	28

Three clock models (the strict, uncorrelated relax and fixed local clock) were used for estimating evolutionary rate

*aa* amino acid

## Discussion

The GSH and Trx systems are two major thiol-dependent antioxidant systems, which are also involved in regulating the redox state of GPxs and TPxs, respectively. Unlike human GPxs, which use GSH as an electron donor, many fungal GPxs prefer Trx over GSH as an electron donor *in vivo*. In this study, we carried out a bioinformatics analysis to understand the basis for the selection of Trx by both spGpx1 and spTpx1. Sequence analysis revealed that unlike human GPxs, which contain a single conserved Cys/ Sec residue (called peroxidatic Cys/ Sec), *S. pombe* and *S. cerevisiae* GPxs all contain two conserved Cys residues called the peroxidatic and resolving Cys residues, respectively. It has been shown that all three *S. cerevisiae* GPxs function as atypical 2-Cys Prxs which form an intramolecular disulfide bond between the two conserved Cys residues and use Trx as an electron donor for recycling of the oxidized peroxidatic Cys residue in vivo (Delaunay et al. 2002, Tanaka et al. 2005, Ohdate et al. 2010). Our analysis predicts that spGpx1 is likely to function as a 2-Cys Prx using Trx as an electron donor to reduce the disulfide bond between Cys36 and Cys82 residues. Molecular modelling of spGpx1 suggests that a conformational change upon Cys36 oxidation may be required to bring these two Cys residues together. This is consistent with the idea that upon oxidation of the peroxidatic Cys residue, a conformational change in the active sites of Prxs is required for the disulfide bond formation between the peroxidatic and resolving Cys residues (Hall et al. 2011, Zhou et al. 2016). However, we cannot predict whether Cys36 and Cys82 residues in spGpx1 form disulfide bond intramolecularly or intermolecularly.

spGpx1 is phylogenetically and functionally distinct from spTpx1. No sequence similarity between spGpx1 and spTpx1 was detected. While both proteins are H_2_O_2_ scavengers, they play different roles in cells. spGpx1 is localized in both the cytosol and mitochondria, and is the predominant peroxidase in stationary phase (Lee et al. 2008). In contrast, spTpx1 is a ROS sensor that transduces the redox signal to the transcription factor Pap1 (Calvo et al. 2013). Unlike spGpx1, spTpx1 is required for the normal aerobic growth of *S. pombe* (Jara et al. 2007). Despite these differences between spGpx1 and spTpx1, our phylogenetic analysis suggests that the two proteins may share a common ancestor and use a similar mechanism for the regeneration of the active-site peroxidatic Cys residue.

## Supplementary Information

**Additional file 1:** **Figure S1.** Phylogenetic tree of GPxs from *S. pombe*, *S. cerevisiae *and humans. The phylogenetic tree was constructed using the MEGA-X software. The evolution history was inferred by using the Maximum Likelihood method and JTT matrix-based model. The tree with the highest log likelihood (-4190.76) is shown. The percentage of trees in which the associated taxa clustered together is shown next to the branches. Initial tree (s) for the heuristic search was obtained automatically by applying Neighbor-Join and BioNJ algorithms to a matrix of pairwise distances estimated using the JTT model, and then selecting the topology with superior log likelihood value. There was a total of 238 positions in the final dataset. **Figure S2.** Phylogenetic tree of TPxs from *S. pombe*, *S. cerevisiae *and humans. The phylogenetic tree was constructed using the MEGA-X software. The evolution history was inferred by using the Maximum Likelihood method and JTT matrix-based model. The tree with the highest log likelihood (-3453.60) is shown. The percentage of trees in which the associated taxa clustered together is shown next to the branches. Initial tree(s) for the heuristic search were obtained automatically by applying Neighbor-Join and BioNJ algorithms to a matrix of pairwise distances estimated using the JTT model, and then selecting the topology with superior log likelihood value. There was a total of 305 positions in the final dataset. **Table S1. **Comparison of spGpx1 with selected candidate fungal GPxs used in the evolutionary analysis. **Table S2. **Comparison of spTpx1 with selected candidiate fungal TPxs used in the evolutionary analysis

## Data Availability

The data sets generated and analyzed during the current study are available on the request from the corresponding author.

## References

[CR1] Bolduc J, Koruza K, Luo T, Malo Pueyo J, Vo TN, Ezeriņa D, Messens J (2021). Peroxiredoxins wear many hats: factors that fashion their peroxide sensing personalities. Redox Biology.

[CR2] Bozonet SM, Findlay VJ, Day AM, Cameron J, Veal EA, Morgan BA (2005). Oxidation of a eukaryotic 2-Cys peroxiredoxin is a molecular switch controlling the transcriptional response to increasing levels of hydrogen peroxide. J Biol Chem.

[CR3] Brigelius-Flohé R, Maiorino M (2013). Glutathione peroxidases. Biochim Biophys Acta.

[CR4] Calvo IA, Boronat S, Domènech A, García-Santamarina S, Ayté J, Hidalgo E (2013). Dissection of a redox relay: H_2_O_2_-dependent activation of the transcription factor Pap1 through the peroxidatic Tpx1-thioredoxin cycle. Cell Rep.

[CR5] Delaunay A, Pflieger D, Barrault MB, Vinh J, Toledano MB (2002). A thiol peroxidase is an H_2_O_2_ receptor and redox-transducer in gene activation. Cell.

[CR6] Do TD, Thi Mai N, Duy Khoa TN, Abol-Munafi AB, Liew HJ, Kim CB, Wong LL (2019). Molecular characterization and gene expression of glutathione peroxidase 1 in tor tambroides exposed to temperature stress. Evol Bioinform Online.

[CR7] Hall A, Nelson K, Poole LB, Karplus PA (2011). Structure-based insights into the catalytic power and conformational dexterity of peroxiredoxins. Antioxid Redox Signal.

[CR8] Horton P, Park KJ, Obayashi T, Fujita N, Harada H, Adams Collier CJ, Nakai K (2007). WoLF PSORT: protein localization predictor. Nucleic Acids Res.

[CR9] Jara M, Vivancos AP, Calvo IA, Moldón A, Sansó M, Hidalgo E (2007). The peroxiredoxin Tpx1 is essential as a H_2_O_2_ scavenger during aerobic growth in fission yeast. Mol Biol Cell.

[CR10] Kim JS, Bang MA, Lee S, Chae HZ, Kim K (2010). Distinct functional roles of peroxiredoxin isozymes and glutathione peroxidase from fission yeast *Schizosaccharomyces**pombe*. BMB Rep.

[CR11] Kong H, Chandel NS (2018). Regulation of redox balance in cancer and T cells. J Biol Chem.

[CR12] Kritsiligkou P, Chatzi A, Charalampous G, Mironov A, Grant CM, Tokatlidis K (2017). Unconventional targeting of a thiol peroxidase to the mitochondrial intermembrane space facilitates oxidative protein folding. Cell Rep.

[CR13] Kumar S, Stecher G, Li M, Knyaz C, Tamura K (2018). MEGA X: molecular evolutionary genetics analysis across computing platforms. Mol Biol Evol.

[CR14] Lee SY, Song JY, Kwon ES, Roe JH (2008). Gpx1 is a stationary phase-specific thioredoxin peroxidase in fission yeast. Biochem Biophys Res Commun.

[CR15] Letunic I, Bork P (2018). 20 years of the SMART protein domain annotation resource. Nucleic Acids Res.

[CR16] Lu J, Holmgren A (2014). The thioredoxin antioxidant system. Free Radic Biol Med.

[CR17] Lubos E, Loscalzo J, Handy DE (2011). Glutathione peroxidase-1 in health and disease: from molecular mechanisms to therapeutic opportunities. Antioxid Redox Signal.

[CR18] Matoušková P, Hanousková B, Skálová L (2018). MicroRNAs as potential regulators of glutathione peroxidases expression and their role in obesity and related pathologies. Int J Mol Sci.

[CR19] Monteiro G, Horta BB, Pimenta DC, Augusto O, Netto LE (2007). Reduction of 1-Cys peroxiredoxins by ascorbate changes the thiol-specific antioxidant paradigm, revealing another function of vitamin C. Proc Natl Acad Sci USA.

[CR20] Nordberg J, Arnér ES (2001). Reactive oxygen species, antioxidants, and the mammalian thioredoxin system. Free Radic Biol Med.

[CR21] Ohdate T, Inoue Y (2012). Involvement of glutathione peroxidase 1 in growth and peroxisome formation in *Saccharomyces cerevisiae* in oleic acid medium. Biochim Biophys Acta.

[CR22] Ohdate T, Kita K, Inoue Y (2010). Kinetics and redox regulation of Gpx1, an atypical 2-Cys peroxiredoxin *Saccharomyces cerevisiae*. FEMS Yeast Res.

[CR23] Paulo E, García-Santamarina S, Calvo IA, Carmona M, Boronat S, Domènech A, Ayté J, Hidalgo E (2014). A genetic approach to study H_2_O_2_ scavenging in fission yeast–distinct roles of peroxiredoxin and catalase. Mol Microbiol.

[CR24] Pedrajas JR, McDonagh B, Hernández-Torres F, Miranda-Vizuete A, González-Ojeda R, Martínez-Galisteo E, Padilla CA, Bárcena JA (2016). Glutathione is the resolving thiol for thioredoxin peroxidase activity of 1-Cys peroxiredoxin without being consumed during the catalytic cycle. Antioxid Redox Signal.

[CR25] Peng S, Cheng M, Huang K, Cui Y, Zhang Z, Guo R, Zhang X, Yang S, Liao X, Lu Y, Zou Q, Shi B (2018). Efficient computation of motif discovery on intel many integrated core (mic) architecture. BMC Bioinformatics.

[CR26] Perkins A, Nelson KJ, Parsonage D, Poole LB, Karplus PA (2015). Peroxiredoxins: guardians against oxidative stress and modulators of peroxide signaling. Trends Biochem Sci.

[CR27] Quinn J, Findlay VJ, Dawson K, Millar JBA, Jones N, Morgan BA, Toone WM (2002). Distinct regulatory proteins control the graded transcriptional response to increasing H_2_O_2_ levels in fission yeast *Schizosaccharomyces pombe*. Mol Biol Cell.

[CR28] Rambaut A, Drummond AJ, Xie D, Baele G, Suchard MA (2018). Posterior summarization in bayesian phylogenetics using tracer 17. Syst Biol.

[CR29] Rhee SG (2016). Overview on peroxiredoxin. Mol Cells.

[CR30] Rhee SG, Kil IS (2017). Multiple functions and regulation of mammalian peroxiredoxins. Annu Rev Biochem.

[CR31] Schieber M, Chandel NS (2014). ROS function in redox signaling and oxidative stress. Curr Biol.

[CR32] Shadel Gerald S, Horvath Tamas L (2015). Mitochondrial ROS signaling in organismal homeostasis. Cell.

[CR33] Sies H, Jones DP (2020). Reactive oxygen species (ROS) as pleiotropic physiological signalling agents. Nat Rev Mol Cell Biol.

[CR34] Tairum CA, de Oliveira MA, Horta BB, Zara FJ, Netto LE (2012). Disulfide biochemistry in 2-cys peroxiredoxin: requirement of Glu50 and Arg146 for the reduction of yeast Tsa1 by thioredoxin. J Mol Biol.

[CR35] Tairum CA, Santos MC, Breyer CA, Geyer RR, Nieves CJ, Portillo-Ledesma S, Ferrer-Sueta G, Toledo JC, Toyama MH, Augusto O, Netto LE, de Oliveira MA (2016). Catalytic Thr or Ser residue modulates structural switches in 2-Cys peroxiredoxin by distinct mechanisms. Sci Rep.

[CR36] Tanaka T, Izawa S, Inoue Y (2005). GPX2, encoding a phospholipid hydroperoxide glutathione peroxidase homologue, codes for an atypical 2-Cys peroxiredoxin in *Saccharomyces cerevisiae*. J Biol Chem.

[CR37] Tosatto SC, Bosello V, Fogolari F, Mauri P, Roveri A, Toppo S, Flohé L, Ursini F, Maiorino M (2008). The catalytic site of glutathione peroxidases. Antioxid Redox Signal.

[CR38] Ukai Y, Kishimoto T, Ohdate T, Izawa S, Inoue Y (2011). Glutathione peroxidase 2 in *Saccharomyces cerevisiae* is distributed in mitochondria and involved in sporulation. Biochem Biophys Res Commun.

[CR39] Vivancos AP, Castillo EA, Biteau B, Nicot C, Ayté J, Toledano MB, Hidalgo E (2005). A cysteine-sulfinic acid in peroxiredoxin regulates H_2_O_2_-sensing by the antioxidant Pap1 pathway. Proc Natl Acad Sci U S A.

[CR40] Winterbourn CC (2008). Reconciling the chemistry and biology of reactive oxygen species. Nat Chem Biol.

[CR41] Wood ZA, Poole LB, Karplus PA (2003). Peroxiredoxin evolution and the regulation of hydrogen peroxide signaling. Science.

[CR42] Wood ZA, Schröder E, Robin Harris J, Poole LB (2003). Structure, mechanism and regulation of peroxiredoxins. Trends Biochem Sci.

[CR43] Xia X, Hua C, Xue S, Shi B, Gui G, Zhang D, Wang X, Guo L (2016). Response of selenium-dependent glutathione peroxidase in the freshwater bivalve Anodonta woodiana exposed to 2,4-dichlorophenol,2,4,6-trichlorophenol and pentachlorophenol. Fish Shellfish Immunol.

[CR44] Zhang Y (2008). I-TASSER server for protein 3D structure prediction. BMC Bioinformatics.

[CR45] Zhang R, Drummond A (2020). Improving the performance of Bayesian phylogenetic inference under relaxed clock models. BMC Evol Biol.

[CR46] Zhang WJ, He YX, Yang Z, Yu J, Chen Y, Zhou CZ (2008). Crystal structure of glutathione-dependent phospholipid peroxidase Hyr1 from the yeast *Saccharomyces cerevisiae*. Proteins.

[CR47] Zhou S, Sorokina EM, Harper S, Li H, Ralat L, Dodia C, Speicher DW, Feinstein SI, Fisher AB (2016). Peroxiredoxin 6 homodimerization and heterodimerization with glutathione S-transferase pi are required for its peroxidase but not phospholipase A2 activity. Free Radic Biol Med.

