# Supplementary material for: Basis for using thioredoxin as an electron donor by Schizosaccharomyces pombe Gpx1 and Tpx1
Source: AMB Express. 2022 Apr 11;12:41. doi: 10.1186/s13568-022-01381-2 (PMC9001804; doi:10.1186/s13568-022-01381-2)
Supplement: Supplementary file 1 — Additional file 1: Figure S1. Phylogenetic tree of GPxs from S. pombe, S. cerevisiae and humans. The phylogenetic tree was constructed using the MEGA-X software. The evolution history was inferred by using the Maximum Likelihood method and JTT matrix-based model. The tree with the highest log likelihood (-4190.76) is shown. The percentage of trees in which the associated taxa clustered together is shown next to the branches. Initial tree (s) for the heuristic search was obtained automatically by applying Neighbor-Join and BioNJ algorithms to a matrix of pairwise distances estimated using the JTT model, and then selecting the topology with superior log likelihood value. There was a total of 238 positions in the final dataset. Figure S2. Phylogenetic tree of TPxs from S. pombe, S. cerevisiae and humans. The phylogenetic tree was constructed using the MEGA-X software. The evolution history was inferred by using the Maximum Likelihood method and JTT matrix-based model. The tree with the highest log likelihood (-3453.60) is shown. The percentage of trees in which the associated taxa clustered together is shown next to the branches. Initial tree(s) for the heuristic search were obtained automatically by applying Neighbor-Join and BioNJ algorithms to a matrix of pairwise distances estimated using the JTT model, and then selecting the topology with superior log likelihood value. There was a total of 305 positions in the final dataset. Table S1. Comparison of spGpx1 with selected candidate fungal GPxs used in the evolutionary analysis. Table S2. Comparison of spTpx1 with selected candidiate fungal TPxs used in the evolutionary analysis [file 13568_2022_1381_MOESM1_ESM.pdf]

Basis for using thioredoxin as an electron donor by *Schizosaccharomyces pombe* Gpx1 and Tpx1

Fawad Ahmad[1#], Muhammad Faizan Latif[1#], Ying Luo[1] and Ying Huang[1]*

[1]Jiangsu Key Laboratory for Microbes and Genomics, Department of Microbiology, School of Life Sciences, Nanjing Normal University, 1 Wenyuan Road, Nanjing 210023, China

[#]These authors contributed equally to this work.

Corresponding author:

Ying Huang, PhD.

Jiangsu Key Laboratory for Microbes and Genomics

Department of Microbiology

School of Life Sciences

Nanjing Normal University

1 Wenyuan Road,

Nanjing 210023, China

Tel: +86025-85891263

Email: yhuang@njnu.edu.cn

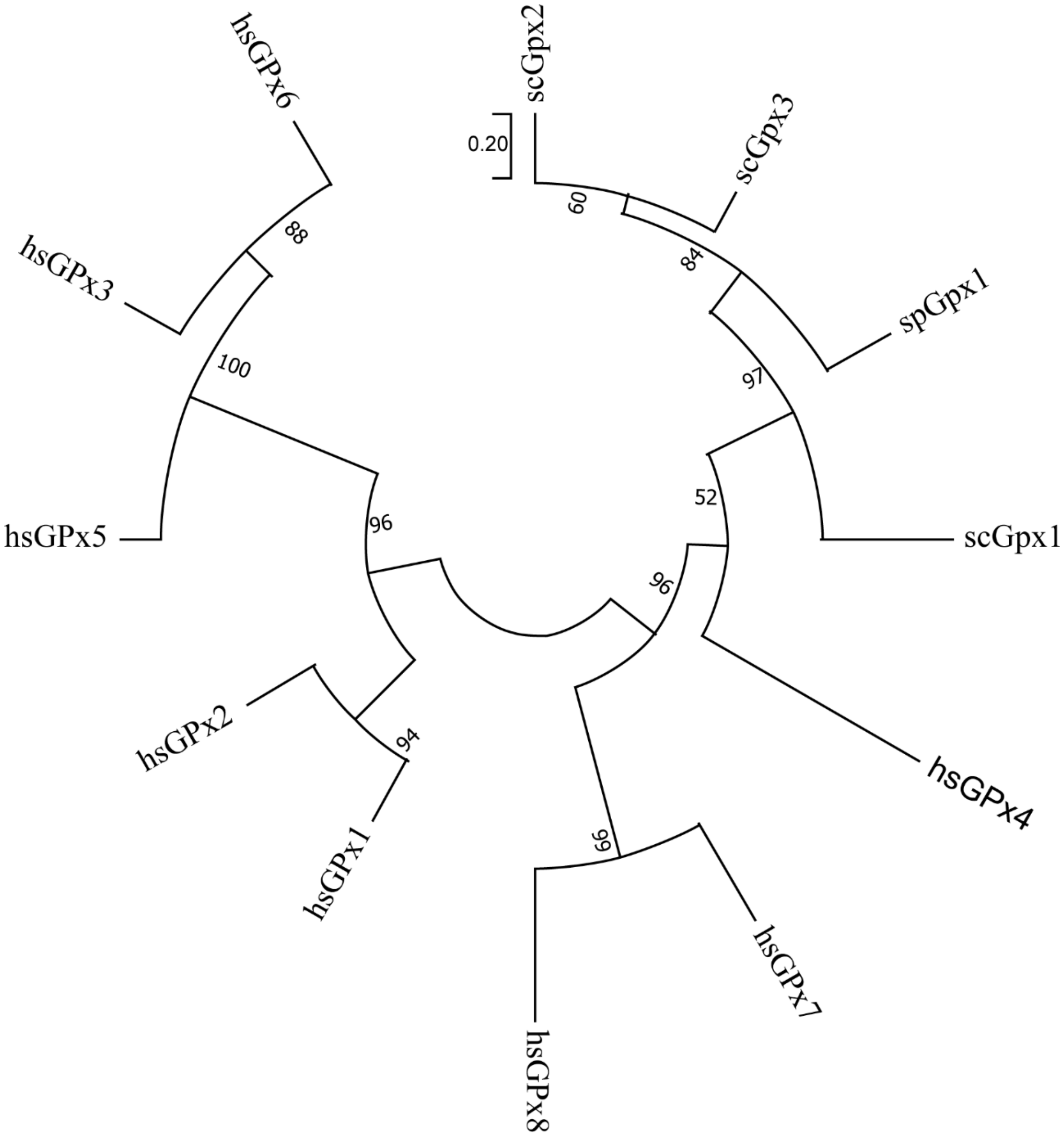

Fig. S1 Phylogenetic tree of GPxs from *S. pombe, S. cerevisiae* and humans. The phylogenetic tree was constructed using the MEGA-X software. The evolution history was inferred by using the Maximum Likelihood method and JTT matrix-based model. The tree with the highest log likelihood (-4190.76) is shown. The percentage of trees in which the associated taxa clustered together is shown next to the branches. Initial tree (s) for the heuristic search was obtained automatically by applying Neighbor-Join and BioNJ algorithms to a matrix of pairwise distances estimated using the JTT model, and then selecting the topology with superior log likelihood value. There was a total of 238 positions in the final dataset.

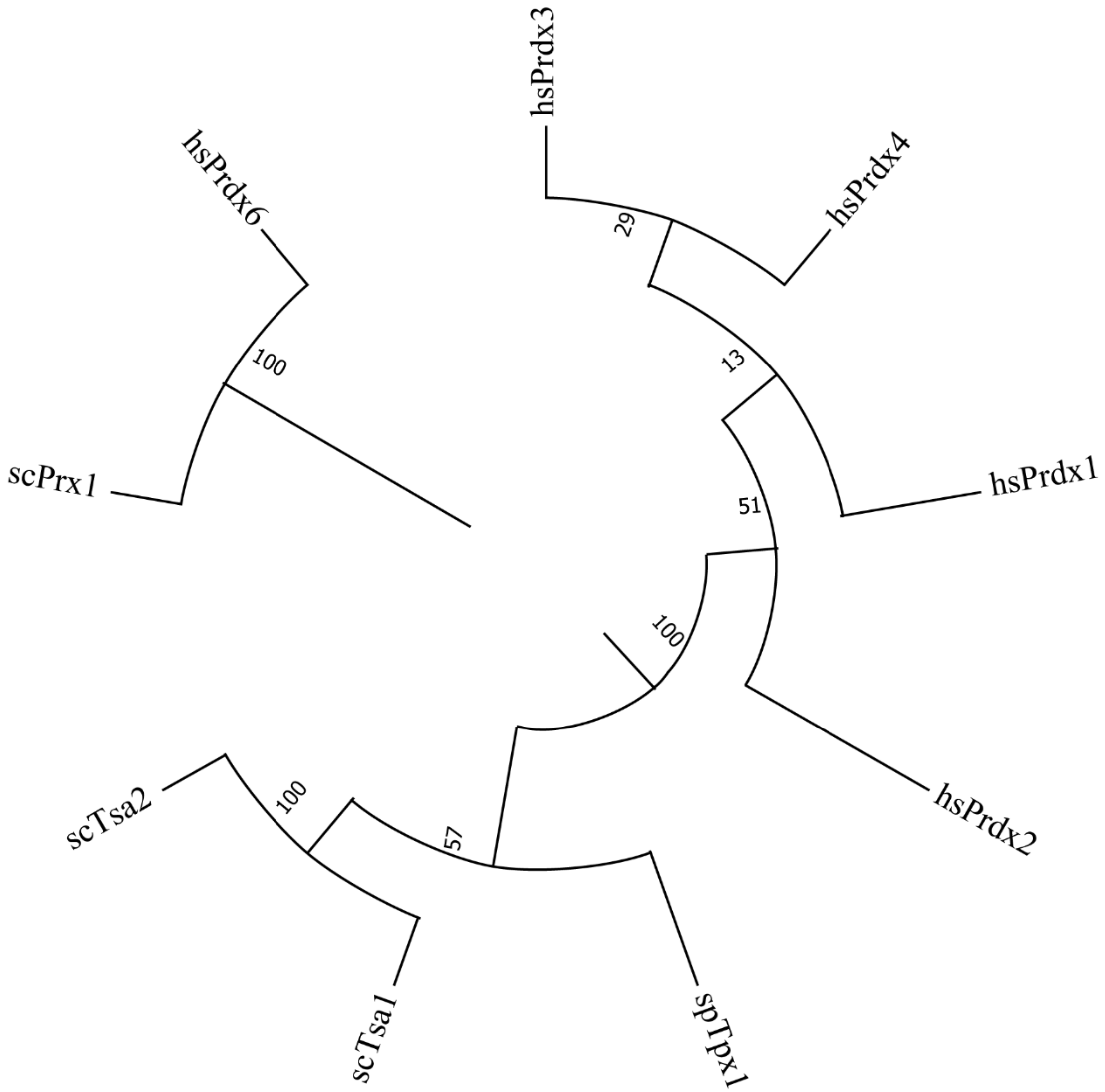

Fig. S2 Phylogenetic tree of TPxs from *S. pombe, S. cerevisiae* and humans. The phylogenetic tree was constructed using the MEGA-X software. The evolution history was inferred by using the Maximum Likelihood method and JTT matrix-based model. The tree with the highest log likelihood (-3453.60) is shown. The percentage of trees in which the associated taxa clustered together is shown next to the branches. Initial tree(s) for the heuristic search were obtained automatically by applying Neighbor-Join and BioNJ algorithms to a matrix of pairwise distances estimated using the JTT model, and then selecting the topology with superior log likelihood value. There was a total of 305 positions in the final dataset.

Table S1 Comparison of spGpx1 with selected candidate fungal GPxs used in the evolutionary analysis

	spGpx1		
	Accession No.	Identity (%)	Positive (%)
S. cerevisiae Gpx1	GFP67937.1	56	72
S. cerevisiae Gpx2	GFP72072.1	66	81
S. cerevisiae Gpx3	P40581.1	73	83
A. nidulans PHGPx	CBF83884.1	62	77
E. gossypii Gpx1	NP_985509.1	68	82
N. crassa Gpx3	EAA28683.2	57	75
B. cinereal Gpx3	EMR87195.1	60	77
C. albicans Hyr1	RLP65748.1	69	80
C. albicans Gpx1	AOW30032.1	54	75
C. albicans Gpx2	AOW30030.1	60	77
C. albicans Gpx3	KAG8204604.1	49	68
C. neoformans Gpx1	OXH70166.1	57	72
H. capsulatum Gpx1	QSS63045.1	61	79
C. venosus Gpx1	RPA91107.1	63	79
A. nigricans Gpx1	TGZ81662.1	58	77
A. immerses Gpx1	RPA88258.1	62	75
S. paradoxus Gpx3	XP_033767077.1	73	83
S. cryophilus Gpx1	XP_013023707.1	85	94
R. solani Gpx1	CUA68428.1	65	79
C. ramicola Gpx2	KAG8729849.1	65	79
T. irregularis Gpx1	KAG9033083.1	61	78
R. microspores Gpx1	ORE19597.1	57	76
A. ossiformis Gpx2	KAF7727402.1	59	74
C. cucurbitarum Gpx2	OBZ89072.1	57	76
G. prolifera Gpx1	KXS21034.1	60	80
B. helices Gpx3	RKO83242.1	58	70
C. protostelioides Gpx1	RKO95323.1	53	70

Table S2 Comparison of spTpx1 with selected candidiate fungal TPxs used in the evolutionary analysis

	spTpx1		
	Accession No.	Identity (%)	Similarity (%)
S. cerevisiae Tsa1	KAF4004212.1	65	78
S. cerevisiae Tsa2	KZV12693.1	62	78
S. cerevisiae Prx1	GFP66757.1	32	48
A. nidulans Prx1	CBF85378.1	32	50
E. gossypii Tsa1	NP_985168.1	62	77
B. cinerea Prx3	XP_024553582.1	31	48
C. albicans Tsa1	KAF6072083.1	65	81
C. neoformans Tsa1	XP_571871.1	61	75
H. capsulatum Tsa1	AAK54753.1	32	51
C. venosus Tsa1	RPB04080.1	65	81
A. nigricans Tsa1	TGZ80469.1	65	83
A. immersus Tsa1	RPA74368.1	62	72
S. paradoxus Tsa1	XP_033768287.1	67	79
S. paradoxus Tsa2	XP_033765666.1	63	78
S. paradoxus Prx1	XP_033764536.1	32	48
S. cryophilus Tpx1	XP_013023818.1	89	93
R. solani Prx1	CUA76735.1	65	77
C. ramicola Tsa1	QRV91064.1	64	79
T. irregularis Tpx1	KAG8906759.1	63	75
R. microsporus Tsa1	KAG8906759.1	64	78
A. ossiformis Tpx1	KAF7725453.1	63	79
C. cucurbitarum Prx1	OBZ87541.1	65	80
C. cucurbitarum Prx2	OBZ87058.1	53	74
C. cucurbitarum Prx3	OBZ91928.1	33	50
G. prolifera Tsa1	KXS15864.1	57	74
B. helicus TXNPx	RKO92741.1	59	75
C. protostelioides Tsa1	RKO96964.1	51	65
